# Supplementary material for: Partial restoration of immune response in Hepatitis C patients after viral clearance by direct-acting antiviral therapy
Source: PLoS One. 2021 Jul 9;16(7):e0254243. doi: 10.1371/journal.pone.0254243 (PMC8270431; doi:10.1371/journal.pone.0254243)
Supplement: S1 Table — Age, gender, HCV genotype, previous Treatment, DAA regimens, fibroscan, transaminase enzymes (AST, ALT), APRI, FIB-4 and AFP levels were taken at baseline and FUW12. (PDF) [file pone.0254243.s001.pdf]

PATIENT ID	Age	Gender	HCV Genotype	Previous Treatment	Treatment	BASELINE						FUW12					
						Fibroscan	AST	ALT	APRI	FIB-4	AFP	Fibroscan	AST	ALT	APRI	FIB-4	AFP
**1**	61	F	G1b	Naive	SOF/LDV	7.9	51	59	0.7	1.9	4.2	3.8	20	14	0.3	1.7	4.7
**2**	51	M	G1b	PegIFN/RBV	SOF/LDV	8.3	186	321	2.0	2.8	3.8	4.7	30	19	0.3	1.7	1.0
**3**	53	F	G1b	PegIFN/RBV	SOF/LDV	22	117	138	2.6	4.0	9.4	9.9	30	24	0.6	2.3	3.9
**4**	60	F	G1b	Naive	SOF/LDV	17.5	76	93	1.4	3.0	8.4	7	23	18	0.3	1.6	6.1
**5**	75	F	G1b	Naive	SOF/LDV	9.8	36	25	0.7	3.5	4.2	NA	18	12	0.3	2.4	3.4
**6**	60	M	G1b	PegIFN/RBV	SOF/LDV +RBV	8.6	32	36	0.2	1.2	1.6	3.2	20	16	0.2	1.2	1.2
**7**	79	F	G1b	Naive	SOF/LDV	18.4	120	82	3.0	9.1	49.2	12	53	41	1.5	6.5	13.0
**8**	47	M	G1b	PegIFN/RBV	SOF/LDV	23.1	72	137	0.8	1.7	1.7	4.4	27	34	0.3	1.2	1.0
**9**	82	F	G1b	PegIFN/RBV	SOF/LDV	10.9	41	85	0.5	1.4	4.3	8	15	23	0.2	1.1	2.7
**10**	55	F	G1b	Naive	SOF/LDV	18.2	66	114	1.0	1.9	8.6	NA	23	19	0.3	1.4	5.4
**11**	75	F	G1b	Naive	SOF/LDV	7.6	80	86	1.9	5.4	10.1	3.2	32	21	0.6	3.6	4.3
**12**	52	F	G1b	PegIFN/RBV	SOF/LDV	7.4	36	56	0.4	1.1	1.6	5.4	18	15	0.2	1.0	0.6
**13**	41	F	G1b	Naive	SOF/LDV	11.8	29	26	0.5	1.3	4.1	4	19	10	0.3	1.2	2.0
**14**	46	F	G1b	PegIFN/RBV/BOC	SOF/LDV	8.2	25	31	0.3	0.8	1.8	5	21	21	0.2	0.7	1.8
**15**	60	F	G1a	Naive	SOF/LDV	9.1	98	143	1.9	3.3	13.0	5.1	28	18	0.4	2.2	12.5
**16**	49	M	G1a	PegIFN/RBV/BOC	SOF/LDV +RBV	9.8	42	77	0.8	2.2	2.9	5.4	26	30	0.4	2.0	2.2
**17**	46	F	G1a	Naive	SOF/LDV	7.9	35	37	0.5	1.3	1.7	4.6	19	11	0.2	1.1	0.6
**18**	27	F	G1a	PegIFN/RBV	SOF/LDV	8.3	27	30	0.3	0.6	1.5	NA	13	9	0.2	0.6	2.4
**19**	45	F	G1a	PegIFN/RBV	SOF/LDV +RBV	17.3	97	130	1.4	2.0	15.0	13.4	19	20	0.3	1.1	4.1
**20**	45	M	G1a	Naive	SOF/SMV	48.8	101	75	2.4	6.2	18.7	NA	38	20	0.8	4.2	6.0
**21**	42	F	G1a	PegIFN/RBV	SOF/LDV	3.9	26	26	0.4	1.2	1.0	3.5	15	12	0.2	1.0	1.0
**22**	50	M	G1a	Naive	OBV/PTV/r + DSV + RBV	7.8	44	51	0.7	2.4	1.2	8.5	30	28	0.3	1.6	1.0
**23**	61	M	G1a	Naive	OBV/PTV/r + DSV + RBV	7.3	27	29	0.2	1.4	2.7	NA	14	10	0.1	1.2	3.0
**24**	47	M	G1a	Naive	OBV/PTV/r + DSV + RBV	11.3	58	102	0.5	1.2	1.6	NA	26	30	0.3	1.1	1.8

**25**	64	M	G1a	Naive	EBR/GZR + RBV	6.7	49	76	0.4	1.6	4.0	5.1	24	19	0.2	1.6	1.7
**26**	47	M	G1a	Naive	SOF/LDV +RBV	8.8	25	34	0.2	0.8	5.5	2.7	19	19	0.1	0.8	2.7
**27**	53	M	G1a	Naive	EBR/GZR + RBV	7.3	38	54	0.4	1.3	4.3	4.9	25	25	0.2	1.0	2.0
