# Supplementary material for: Partial restoration of immune response in Hepatitis C patients after viral clearance by direct-acting antiviral therapy
Source: PLoS One. 2021 Jul 9;16(7):e0254243. doi: 10.1371/journal.pone.0254243 (PMC8270431; doi:10.1371/journal.pone.0254243)
Supplement: Attachment — Submitted filename: PONE-D-21-11447 Llorens-Revull et al_Response to Reviewers.docx [file pone.0254243.s002.docx]

Professor Tatsuo Kanda

Academic Editor

*PLOS ONE*

## Barcelona, June 9^th^, 2021

Dear Professor Tatsuo Kanda,

Please find attached our revised manuscript PONE-D-21-11447 entitled "Partial restoration of immune response in Hepatitis C patients after viral clearance by direct-acting antiviral therapy" to be considered for publication in *PLOS ONE*.

First, we wish to express our appreciation for the time and care you and the reviewers have taken to evaluate our manuscript and offer comments for improvement. We have made every effort to include all suggestions in the paper, and provide our point-by-point response to the comments on pages below, addressed journal requirements, and introduced all the changes in the revised version of the manuscript and figures.

We hope that the changes that we introduced in the manuscript are adequate for publication in *PLOS ONE*. Please, let me know if additional changes are required for the paper to be accepted in your excellent journal.

Sincerely yours,

Marta Bes

Journal Requirements:

When submitting your revision, we need you to address these additional requirements.

1. Please ensure that your manuscript meets PLOS ONE's style requirements, including those for file naming. The PLOS ONE style templates can be found at

<https://journals.plos.org/plosone/s/file?id=wjVg/PLOSOne_formatting_sample_main_body.pdf>

<https://journals.plos.org/plosone/s/file?id=ba62/PLOSOne_formatting_sample_title_authors_affiliations.pdf>

**Answer:** We have adjusted the manuscript to meet PLOS ONE style requirements.

2. We note that you have included the phrase “data not shown” in your manuscript. Unfortunately, this does not meet our data sharing requirements. PLOS does not permit references to inaccessible data. We require that authors provide all relevant data within the paper, Supporting Information files, or in an acceptable, public repository. Please add a citation to support this phrase or upload the data that corresponds with these findings to a stable repository (such as Figshare or Dryad) and provide and URLs, DOIs, or accession numbers that may be used to access these data. Or, if the data are not a core part of the research being presented in your study, we ask that you remove the phrase that refers to these data.

**Answer:** We have eliminated the phases with “data not shown” or included the data in the text.

3. Have the authors made all data underlying the findings in their manuscript fully available?

The PLOS Data policy requires authors to make all data underlying the findings described in their manuscript fully available without restriction, with rare exception (please refer to the Data Availability Statement in the manuscript PDF file). The data should be provided as part of the manuscript or its supporting information, or deposited to a public repository. For example, in addition to summary statistics, the data points behind means, medians and variance measures should be available. If there are restrictions on publicly sharing data e.g. participant privacy or use of data from a third party those must be specified.

**Answer to points 2 and 3:** All relevant data underlying the findings have been included as Figure 4B, lines 340-344.

4. Review Comments to the Author

Reviewer #1: Meritxell Llorens-Revull et al. reported that, in chronically infected patients, HCV elimination by DAA treatment leads to partial restoration of HCV-specific immune responses. They also mentioned that proliferative capacity of HCV-specific CD4+ and CD8+ T cells is recovered after DAA’s therapies, although they did not examine those from the controls.

1. In abstract section, authors used “HCV CD8+ and CD4+ specific T-cells”. What does it mean “HCV specific-immune response”?

**Answer:** We apologize for the lack of clarity. Our results demonstrated that DAA treatment lead to the partial reversion of CD4+ T cell exhaustion and the restoration of the proliferative capacity of HCV-specific CD4+ and CD8+ T cells. Then, we have edited the abstract section to explain these results more clearly as “… to partial reversion of CD4+ T cell exhaustion.” See line 57 of the clean version.

2. Authors include HCV GT1a and GT1b patients. Authors used several different regimens of DAA treatments. Is there any difference?

**Answer:** We thank reviewer#1 for raising this question. We have included the information in lines 390-391 in the clean version: “All patients achieved SVR irrespective of the specific DAA treatment.” Unfortunately, statistical analysis between different DAA treatments cannot be performed due to the small number of patients treated with DAAs other than sofosbuvir+ledipasvir.

3. “HCV-specific 154 CD4+ and CD8+ antigen-specific T cell responses” The 28 and 98 peptides (15–19-mers with 11–12 amino acid (aa) overlaps) spanning the core and nonstructural protein 3 (NS3), respectively, of HCV subtype 1a and 1b were obtained through the NIH Biodefense and Emerging Infectious Research Resources Repository, NIAID, NIH (peptide arrays, HCV, H77, core and NS3 proteins, NR-3737, NR-3747, NR-3752, NR-37452). H77 is a HCV GT1a strain. What strain did you use for HCVGT1b?

**Answer:** We have added the required information in line 170 of the clean version. The HCVGT1b strain used was J4.

4. In Discussion section, authors should refer the following reference: Sasaki R, et al. Rapid hepatitis C virus clearance by antivirals correlates with immune status of infected patients. J Med Virol. 2019 Mar; 91(3):411-418. doi: 10.1002/jmv.25310. PMID: 30192392. Please discuss more and mention the different findings from them.

**Answer:** We thank the reviewer#1 for this comment. We have found this reference very interesting and commented the results in the discussion. See lines 391-394 “HCV clearance was associated with decreased levels of liver inflammation and fibrosis markers, which correlates with the reduction of liver fibrosis related cytokines after HCV eradication by DAA treatment observed by Sasaki et al(35)” and also lines 441-444 “Interestingly, Sasaki et al. reported higher levels of pro-inflammatory cytokines, including IFN- Ɣ and IL-2, in serum of rapid virological responders as compared with end of treatment responders, suggesting that enhanced host immune status may contribute in HCV clearance(35)”

5. Authors should describe clinical courses of all patients in detail.

**Answer:** We have added new data related to liver inflammation and fibrosis. In addition, we have added a new Table as supplementary information (S1 Table), which includes a comprehensive overview of the clinical parameters at baseline and FUW12 of all patients. Quoted in line 115.

Reviewer #2: The authors, Meritxell Llorens-Revull. et al., report partial restoration of immune response in Hepatitis C patients after viral clearance by direct-acting antiviral therapy.

They analyzed proliferative capacity of HCV-specific CD4+ and CD8+ using cell surface expression of Programmed cell death protein 1 (PD-1), T cell immunoglobulin and mucin domain-containing protein 3 (TIM-3), and Lymphocyte-activation gene 3 (LAG-3), which have been identified as markers of exhausted T cells.
It's a good point of view to focus on the recovery of the immune response, not just the elimination of the virus.
DAA eliminate the virus at a high rate, so, this study needs to focus on how it relates to carcinogenesis.

*Major comments:*

1. The authors should add, AFP, (L1, L3), DCP, ATX, M2BPGi as a carcinogenesis marker and fibrosis marker the point of baseline, W4, EOT and FUW12.

**Answer:** We thank the reviewer#2 for this comment that significantly improved results and enriched our discussion. We have made an effort to report the clinical and biochemical analytical parameters suggested by reviewer #2. We have measured the AFP levels at baseline and FUW12 and include results of other markers of liver inflammation and fibrosis, such as Fibrosis-4 (FIB-4) index, transaminases, and AST-to-platelet ratio index (APRI). Results are shown in figure 5, described in lines 361-369, and values are shown as S1 Table (supplementary material). The added results have enriched discussion focussing in carcinogenesis. See discussion lines 388-394, 398-401 and 404-406.

2. If possible, indicate the status of subsequent cancer rates Due to the difference in partial restoration of immune response.

**Answer:** We thank reviewer#2 for raising up this interesting question. Of note, we have found 2 patients that recently developed hepatocellular carcinoma (HCC). This is a very important finding because none of our patients developed HCC after 50 months of follow-up, however, when re-assessing the clinical outcomes of each patient to answer reviewer’s questions, the cumulative HCC rate after 60 months of follow-up had increased up to 7.4%. See material and methods section; lines 127-128 and discussion section; lines 396-398.

3. The immune system deteriorates with age, but how does this compare with age?

**Answer:** This is a very interesting question that is now considered in the results and discussion. We have found that age has a significant impact on the capacity to reverse CD4 T cell exhausted phenotype. See Figure 1D and lines 252-253, 268-271, 398-404, lines 426-431 and lines 434-435 in discussion section.

*Minor comments:*

1. The author should confirm Table1 for confusion between product names and generic names.

**Answer:** Table 1 has been corrected, removing all product names and referring generic names.
